# Aldosterone-induced salt appetite requires HSD2 neurons

**DOI:** 10.1172/jci.insight.175087

**Published:** 2024-12-06

**Authors:** Silvia Gasparini, Lila Peltekian, Miriam C. McDonough, Chidera J.A. Mitchell, Marco Hefti, Jon M. Resch, Joel C. Geerling

**Affiliations:** 1 Department of Neurology,; 2 Department of Neuroscience and Pharmacology,; 3 Department of Pathology, and; 4 Iowa Neuroscience Institute, University of Iowa, Iowa City, Iowa, USA.

**Keywords:** Endocrinology, Neuroscience, Behavior, Mouse models, Neuroendocrine regulation

## Abstract

Excessive aldosterone production increases the risk of heart disease, stroke, dementia, and death. Aldosterone increases both sodium retention and sodium consumption, and increased sodium consumption may worsen end-organ damage in patients with aldosteronism. Preventing this increase could improve outcomes, but the behavioral mechanisms of aldosterone-induced sodium appetite remain unclear. In rodents, we previously identified aldosterone-sensitive neurons, which express the mineralocorticoid receptor and its prereceptor regulator, 11-β-hydroxysteroid dehydrogenase 2 (HSD2). In the present study, we identified HSD2 neurons in the human brain and then used a mouse model to evaluate their role in aldosterone-induced salt intake. First, we confirmed that dietary sodium deprivation increases aldosterone production, salt intake, and HSD2 neuron activity. Next, we showed that continuous chemogenetic stimulation of HSD2 neurons causes a large and specific increase in salt intake. Finally, we used dose-response studies and genetically targeted ablation of HSD2 neurons to show that these neurons are necessary for aldosterone-induced salt intake. Identifying HSD2 neurons in the human brain and establishing their necessity for aldosterone-induced salt intake in mice improves our understanding of appetitive circuits and highlights this small cell population as a therapeutic target for moderating dietary sodium.

## Abstract 2

We identify aldosterone-sensitive HSD2 neurons in the human brain and show that these neurons are required for aldosterone-induced salt appetite in mice.

## Graphical abstract

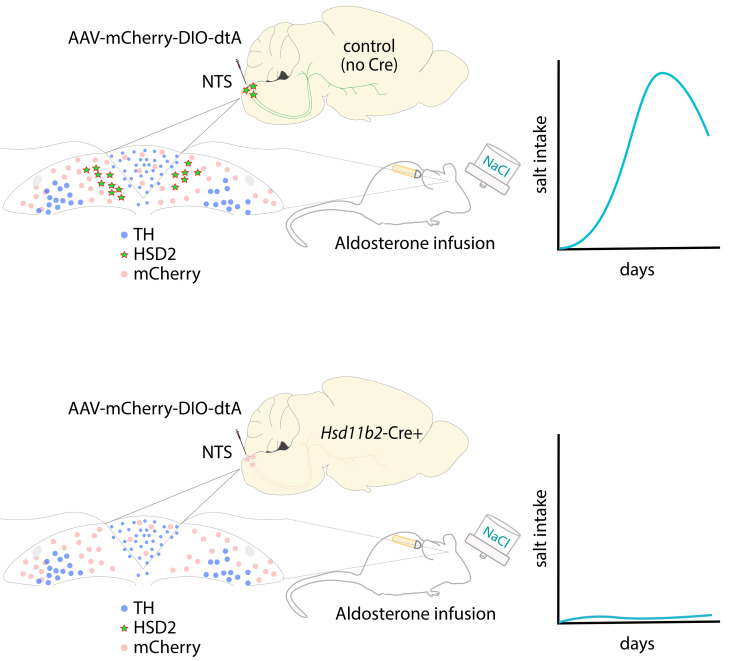

## Introduction

Aldosterone regulates cardiovascular function and electrolyte homeostasis. Production of this steroid hormone rises during hypovolemic states, including prolonged sodium deprivation, and its primary function is to maintain blood volume by increasing sodium reabsorption (1, 2).

Many patients with hypertension have primary aldosteronism, a condition characterized by excessive production of aldosterone (3, 4). Relative to others with hypertension, patients with aldosteronism suffer from stroke, myocardial infarction, atrial fibrillation, and heart failure at several-fold higher rates (5–7) and have moderately elevated rates of diabetes and dementia (8–10).

The reasons for these risk discrepancies are not fully understood, and an aspect that has received little attention is salt consumption. Aldosterone increases sodium appetite in rats (11, 12); furthermore, despite their excessive sodium retention, patients with aldosteronism occasionally report salt cravings (13–15), and those with aldosterone-secreting adenomas consume the most sodium (16). Excessive salt intake is particularly problematic in this patient population because sodium may worsen the severity of cardiac complications, including left ventricular hypertrophy (17–20). Decreasing salt intake reduces blood pressure and may also reduce cardiovascular complications (18–22), so it may help to determine and then disrupt the mechanism of aldosterone-induced salt appetite.

Aldosterone selectively acts in cells that express the enzyme 11-β-hydroxysteroid dehydrogenase type 2 (HSD2) and the mineralocorticoid receptor (MR). HSD2 metabolizes cortisol and other glucocorticosteroids with MR affinity similar to that of aldosterone (23, 24), sensitizing cells to aldosterone by freeing some MR to bind aldosterone, which circulates at concentrations that are 100-fold lower than cortisol (25–27).

HSD2 expression is limited to 1 population of MR-expressing cells in the rodent brain. These cells, the HSD2 neurons, are located in the nucleus of the solitary tract (NTS) (28). Hypovolemic states, including dietary sodium deprivation, increase aldosterone and activate HSD2 neurons (26, 29–32), and chemogenetic stimulation of HSD2 neurons increases salt consumption (33, 34). This circumstantial evidence suggests that HSD2 neurons mediate aldosterone-induced sodium appetite, but it remains unclear if physiologically relevant levels of aldosterone alter sodium appetite (35, 36); whether HSD2 neurons are required; and if other mammals, particularly humans, have HSD2 neurons.

In this study, we first tested whether the human brainstem contains HSD2 neurons. We then confirmed that dietary sodium deprivation increases aldosterone and HSD2 neuron activity in mice and that activating HSD2 neurons increases sodium appetite. Finally, we identified a dose-response relationship between aldosterone and salt intake; we then ablated HSD2 neurons to test the hypothesis that they are necessary for aldosterone-induced sodium appetite.

## Results

### Human HSD2 neurons.

**Figure 1:**
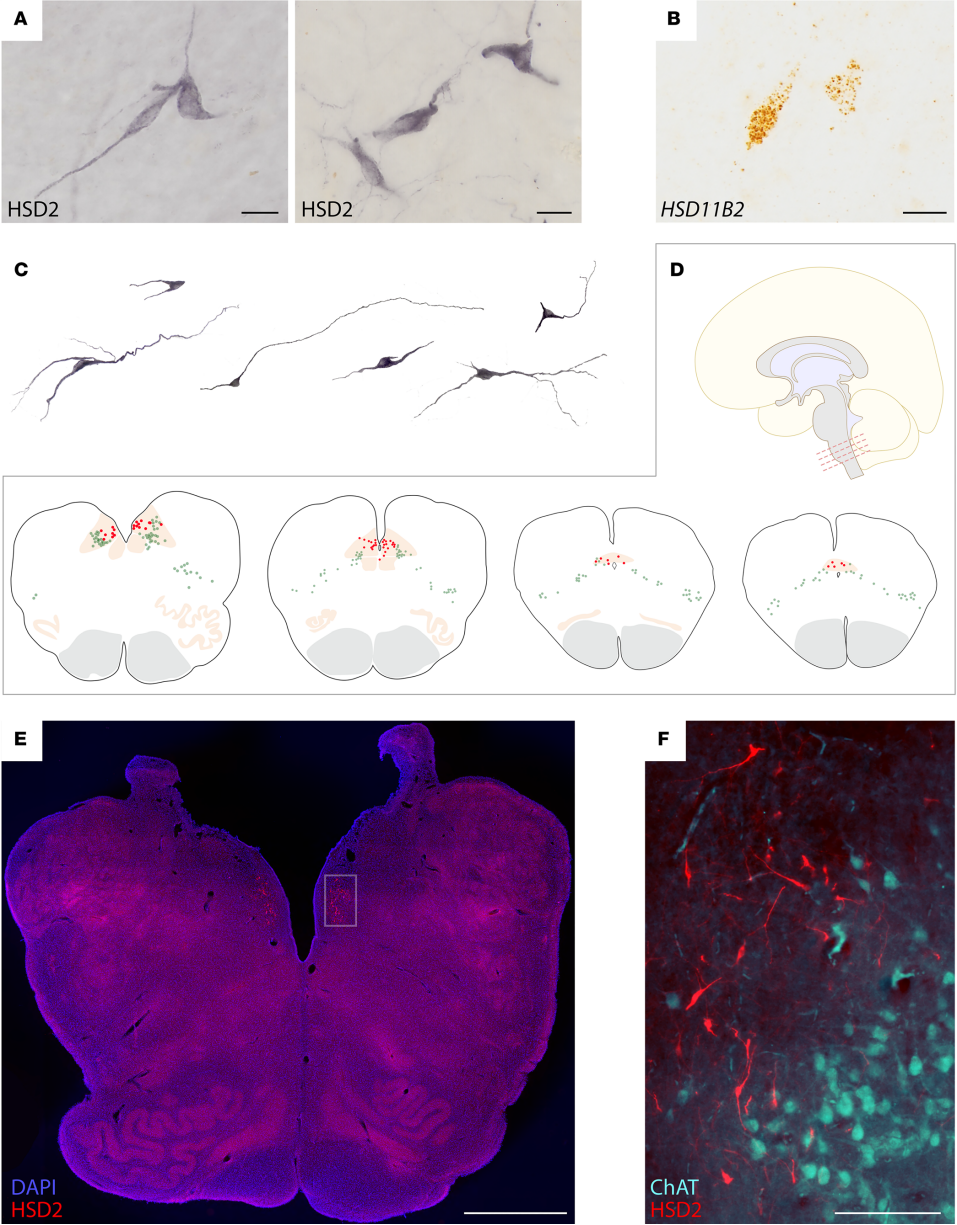
HSD2 (11-β-hydroxysteroid dehydrogenase 2) neurons in the human brain. (**A**) Representative neurons in the nucleus of the solitary tract (NTS) of 2 human cases (nickel-DAB IHC labeling for HSD2 from case MH001 in A1 and case MH005 in A2). (**B**) Representative *HSD11B2* mRNA labeling in the NTS (DAB in situ hybridization from case MH001). (**C**) Additional examples of HSD2 neuronal morphology across several human cases (left to right: MH005, MH001, MH001, MH004, MH001, and MH005). (**D**) Neuroanatomical location and distribution of HSD2 neurons (red dots) and neuromelanin-containing neurons (green dots) at 4 rostrocaudal levels of the medulla oblongata (case MH005). HSD2-immunoreactive neurons are restricted to the caudal-medial NTS, and their distribution runs from the obex of the fourth ventricle back through the caudal commissural NTS immediately rostral to the spinomedullary transition. (**E**) HSD2 immunofluorescence in NTS neurons near the obex of the fourth ventricle. (**F**) Combined HSD2 (red) and choline acetyltransferase (ChAT, blue) immunolabeling reveals the distinction between HSD2 neurons in the medial NTS and cholinergic neurons in the dorsal motor nucleus of the vagus nerve. Scale bars: 20 μm (**A** and **B**), 2 mm (**E**), and 200 μm (**F**).

We began by testing whether there are HSD2 neurons in the human brain. In the caudal NTS, we found IHC labeling for HSD2 protein and in situ hybridization for *HSD11B2* mRNA. As shown in Figure 1, A–C, and Supplemental Figure 1D (supplemental material available online with this article; https://doi.org/10.1172/jci.insight.175087DS1), the location, morphology, and distribution of these neurons closely resembles the HSD2 population in rodents (28, 37). As in rodents, human HSD2 neurons (Figure 1D) lie near the obex of the fourth ventricle and extend caudally, through the commissural NTS, nearly to the spinomedullary transition. Like HSD2 neurons in rats and mice, human HSD2 neurons neighbor a large population of neuromelanin-containing (catecholaminergic) neurons that extends laterally through the NTS and into the medullary reticular formation (Figure 1D). Human HSD2 neurons also neighbor a ventrolateral population of neurons in the dorsal vagal motor nucleus that are immunoreactive for choline acetyltransferase (ChAT; Figure 1, E and F).

Human HSD2 neurons are spindle shaped, with 2 or 3 primary dendrites. The short-axis diameter of each soma averaged 10.9 ± 2.5 μm (range 2.5–18.9 μm; *n* = 278 neurons from *N* = 3 human brainstems). From Abercrombie-corrected rostrocaudal counts of HSD2 neurons across 3 cases, we estimate that the human brainstem contains 958 ± 320 HSD2 neurons (1,252 in case #MH001; 617 in case #MH004; 1,003 in case #MH005), roughly double their number in the rat brainstem (37).

We also detected HSD2-immunoreactive neurons in the medial NTS of pigs, near the obex of the fourth ventricle (Supplemental Figure 1C). Thus, HSD2 neurons have a similar location, distribution, and morphology in several mammalian species, including rodents (mouse and rat), ungulates (pig), and primates (human).

### Sodium deprivation activates mouse HSD2 neurons.

**Figure 2:**
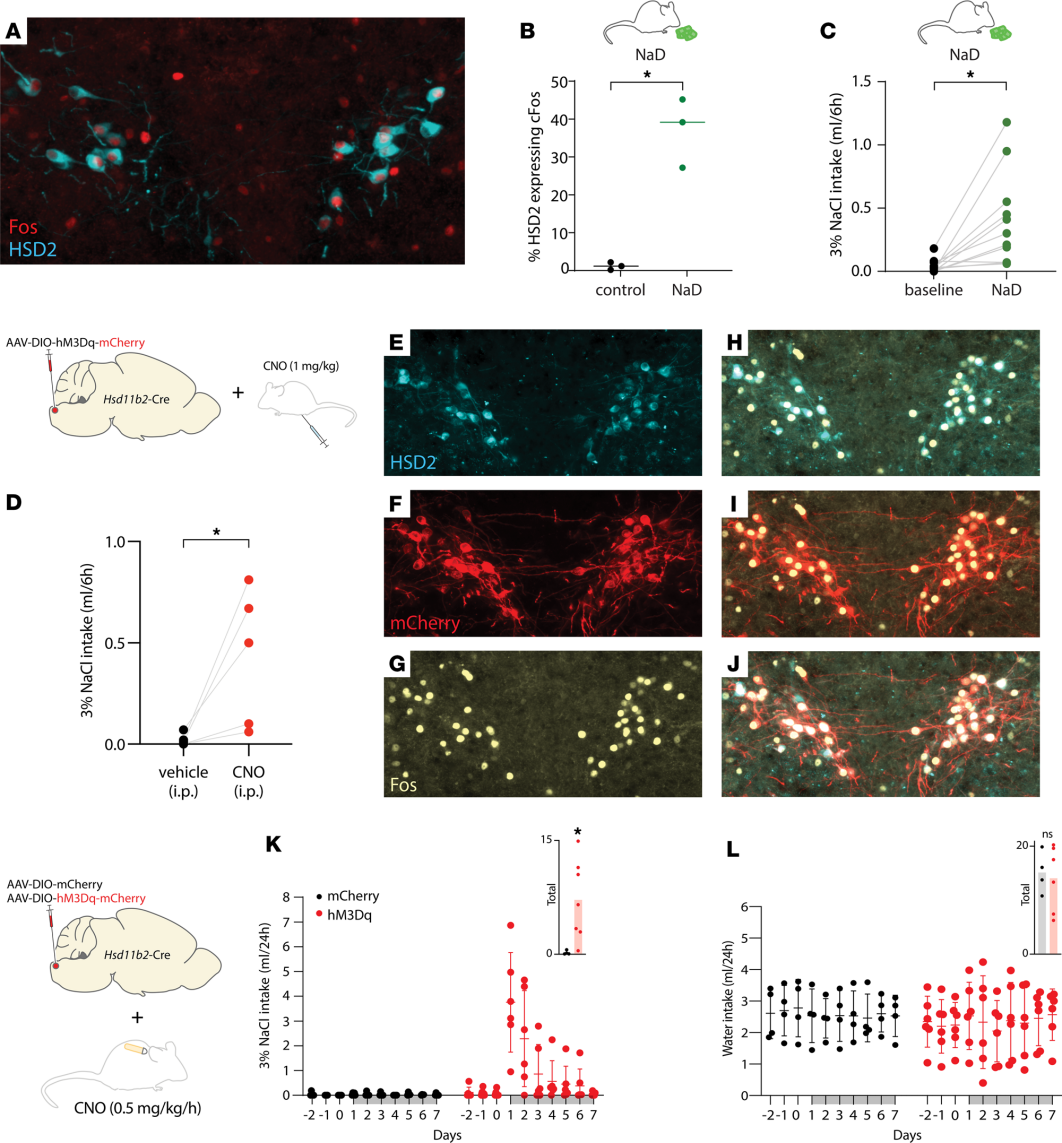
Sodium deprivation and chemogenetic stimulation increase HSD2 neuron activity and salt consumption. (**A** and **B**) Feeding mice low-sodium chow (<0.01% Na, *n* = 3) for 6 days increased the percentage of Fos-immunoreactive HSD2 neurons (*P = 0.0025 versus control mice with ad libitum access to 0.3% Na chow). (**C**) Dietary sodium deprivation also caused mice to consume more saline (*P = 0.0042 versus 3% NaCl intake during previous access to 0.3% Na chow). (**D**) Acute chemogenetic activation of HSD2 neurons by injection of clozapine-N-oxide (CNO, 1 mg/kg i.p.) increased saline intake (*P = 0.0490). (**E**–**J**) CNO (1 mg/kg, 60 minutes prior to perfusion) caused Fos activation of HSD2 neurons expressing Cre-conditional hM3Dq-mCherry. (**K** and **L**) Using an osmotic minipump to continuously infuse CNO (0.5 mg/kg/h) produced a larger increase in saline intake in mice with hM3Dq-mCherry expression (*P = 0.0297 versus mCherry controls) without changing water intake (*P* = 0.7675). Two-tailed t-tests were used for all comparisons in this figure.

Switching rats to a low-sodium diet for 1 week boosts aldosterone production, activates HSD2 neurons, and increases sodium appetite (32, 38, 39). These effects were uncharacterized in mice, so we first assessed the effect of sodium deprivation on HSD2 neurons and sodium appetite. Switching mice to low-sodium chow (<0.01% Na) increased the proportion of HSD2 neurons expressing Fos, a neuronal activity marker (Figure 2, A and B), and consumption of 3% NaCl (Figure 2C). Thus, as in rats, dietary sodium deprivation activates HSD2 neurons and increases sodium appetite in mice.

### HSD2 neurons increase saline intake.

Next, we extended previous reports that stimulating HSD2 neurons increases sodium appetite (33, 34). First, we used *Hsd11b2*-Cre mice to express the excitatory designer receptor exclusively activated by designer drug (DREADD) hM3Dq-mCherry in HSD2 neurons. In these mice, we confirmed that injecting the hM3Dq ligand clozapine-N-oxide (CNO, 1 mg/kg i.p.) activates HSD2 neurons (Figure 2, E–J) and increases saline consumption (Figure 2D). Next, we infused CNO continuously (0.5 mg/kg/h) in mice with either hM3Dq-mCherry or mCherry expressed in HSD2 neurons. Continuous CNO infusion had a larger, hM3Dq-specific effect (Figure 2K), increasing saline intake (*P* = 0.0297) without altering water intake (*P* = 0.7675; Figure 2L).

### Aldosterone dose-dependently increases saline intake.

**Figure 3:**
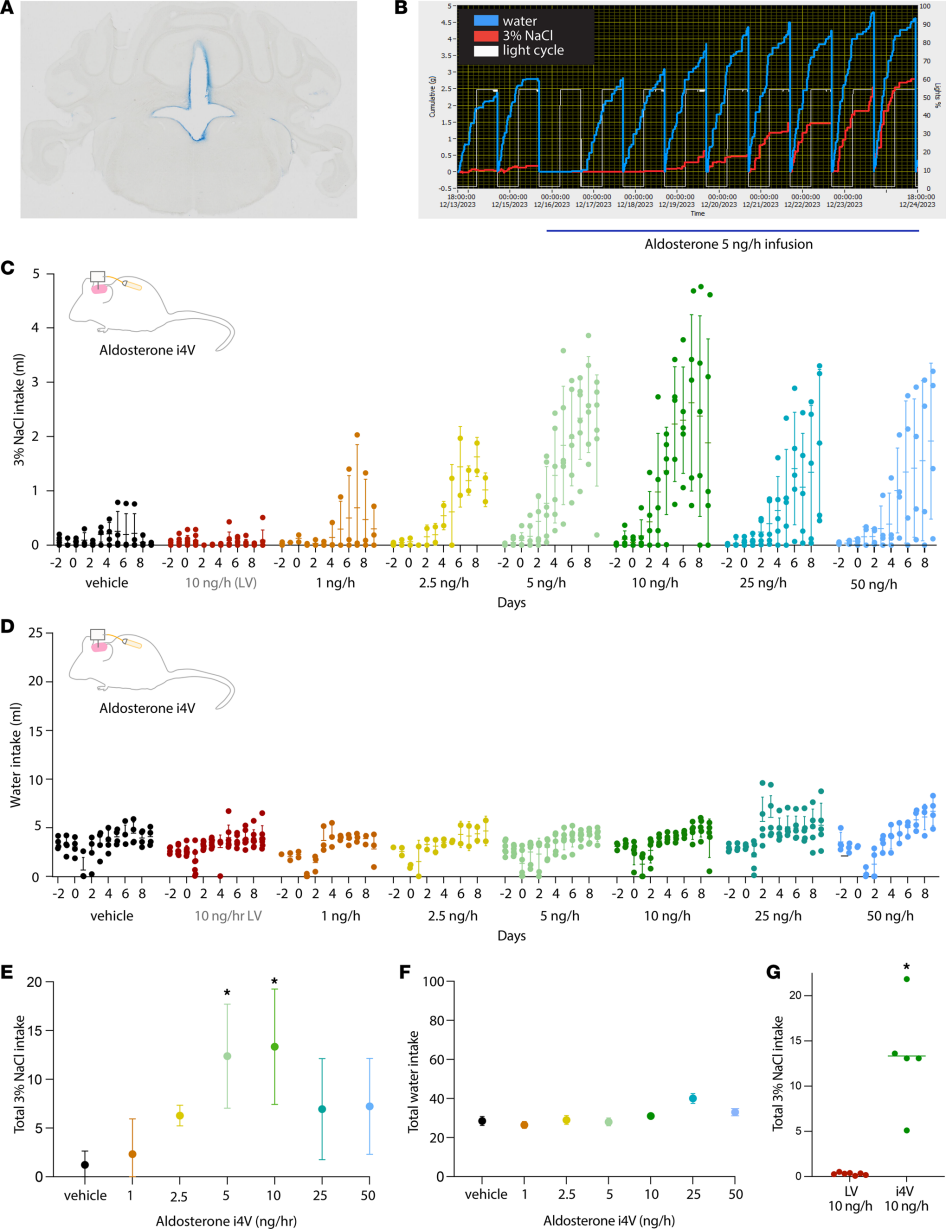
Dose-response relationship between fourth ventricle (i4V) aldosterone infusion and saline intake. (**A**) Example of blue dye passage into in the fourth ventricle, demonstrating patency of the i4V cannula. (**B**) The 3% NaCl (red) and water (blue) intake of a representative mouse receiving 5 ng/h aldosterone. (**C**) The 3% NaCl intake of aldosterone- and vehicle-infused i4V mice, plus a group with infusion of aldosterone into the right lateral ventricle (10 ng/h LV, red). Group mean (horizontal line) and SD (vertical line) are shown for each day, along with individual mouse data. (**D**) Water intake of i4V aldosterone- and vehicle-infused mice, plus a group with 10 ng/h aldosterone infusion into the LV (red). (**E** and **F**) Total (9d) 3% NaCl (**E**) and water (**F**) intake of all i4V groups (asterisks indicate *P* < 0.05 by 1-way ANOVA followed by Tukey’s test for multiple comparisons comparing total 3% NaCl or water intake; *P* = 0.020 for 3% NaCl in 5 ng/h group; *P* = 0.0139 for 3% NaCl in 10 ng/h group). (**G**) Total (9d) 3% NaCl intake in mice receiving 10 ng/h aldosterone in LV (red) versus i4V (green; *P = 0.001 by 2-tailed *t* test).

Previous reports that mineralocorticosteroids do not increase sodium appetite in mice (35, 36) did not test aldosterone, which is the primary mineralocorticosteroid hormone. In contrast, continuously infusing aldosterone into the fourth ventricle (i4V) produced voracious saline intake in rats (11, 12), so we tested whether i4V infusion increases sodium appetite in mice. We began with the infusion rate that had increased saline intake in rats (100 ng/h), but this dose did not increase saline intake, and 3 of 5 mice died unexpectedly or had to be euthanized due to low fluid intake. We therefore performed a series of dose-ranging experiments at lower infusion rates (1–50 ng/h). We recorded 3% NaCl and water intake continuously for 2 days (2d) before through 9d after implanting an osmotic minipump connected to an i4V cannula, and we verified cannula patency by injecting dye through the cannula (Figure 3A).

Vehicle infusion had no effect, but i4V aldosterone caused a dose-dependent rise in saline consumption. Figure 3B shows raw intake data before and during 5 ng/h aldosterone i4V infusion in a representative mouse (3% NaCl, red; water, blue). Saline intake increased gradually and plateaued by day 7 (Figure 3C). We found an inverted U–shaped dose-response relationship, with peak effects at 5–10 ng/h and reduced potency above and below these 2 doses (Figure 3E). Mice receiving 5 or 10 ng/h i4V aldosterone consumed more 3% NaCl than vehicle-infused mice (Figure 3E). The appetitive state induced by i4V aldosterone infusion was selective for saline, as there was no statistically significant effect on water intake at any infusion rate (Figure 3, D and F). Infusing a maximally effective i4V dose (10 ng/h) into the lateral ventricle had no effect on saline intake (*P* = 0.0001 versus i4V; Figure 3G) or water intake (*P* = 0.135), supporting a hindbrain rather than forebrain site of action for aldosterone.

**Figure 4:**
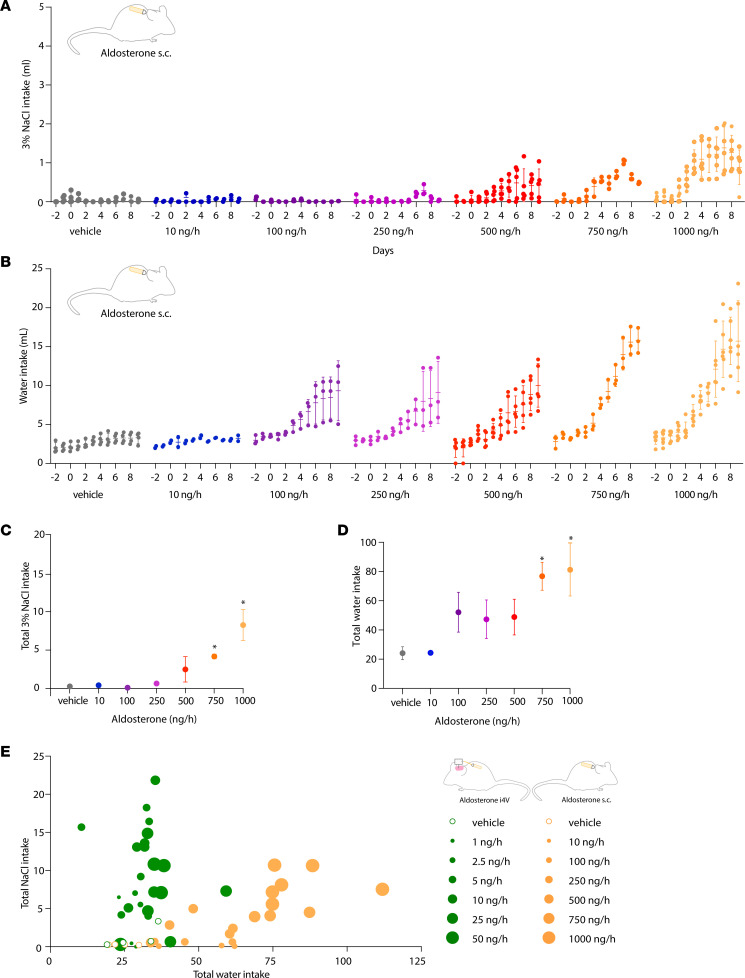
Dose-response relationship between peripheral (s.c.) aldosterone infusion and 3% NaCl intake. (**A**) The 3% NaCl intake of aldosterone- and vehicle-infused mice. Group mean (horizontal line) and SD (vertical line) are shown for each day, along with individual mouse data. (**B**) Water intake of aldosterone- and vehicle-infused mice. (**C** and **D**) Total (9d) 3% NaCl (**C**) and water (**D**) intake across all groups. *P < 0.05 by 1-way ANOVA followed by Tukey’s test for multiple comparisons (*P* = 0.0119 for 3% NaCl in the 750 ng/h group; *P* < 0.0001 for 3% NaCl in the 1000 ng/h group; *P* = 0.0043 for water in the 750 ng/h group; *P* = 0.0006 for water in the 1000 ng/h group). (**E**) Scatter plot shows saline (*y* axis) versus water (*x* axis) intake of every mouse, with dot size indicating rate and color indicating route of infusion (i4V green; s.c. orange).

We next characterized the effect of peripheral infusion, testing a range of infusion rates to reproduce plasma aldosterone levels reported after dietary sodium deprivation, hyperkalemia, kidney disease, and aldosterone-secreting adenomas (32, 40–42). We again found dose-dependent effects, but the maximally effective i4V infusion rate had no effect (Figure 4A), and peripheral infusion was 100-fold less potent (Figure 4C). Unlike the inverted U–shaped response to i4V infusion, the largest peripheral infusion rate (1,000 ng/h) produced the maximum behavioral response. Infusing 750 ng/h had a smaller effect than 1,000 ng/h (*P* = 0.0040), and both 750 ng/h and 1,000 ng/h aldosterone infusion induced significantly more saline intake than vehicle (*P* = 0.0119 and *P* < 0.0001). In contrast to the salt specificity of i4V infusion, peripheral aldosterone infusion increased water intake at all rates above 10 ng/h (Figure 4, B and D). Plotting total saline versus water intake for every mouse (Figure 4E) confirmed that i4V infusion selectively increased saline intake, while peripheral infusion increased both saline and water intake.

**Figure 5:**
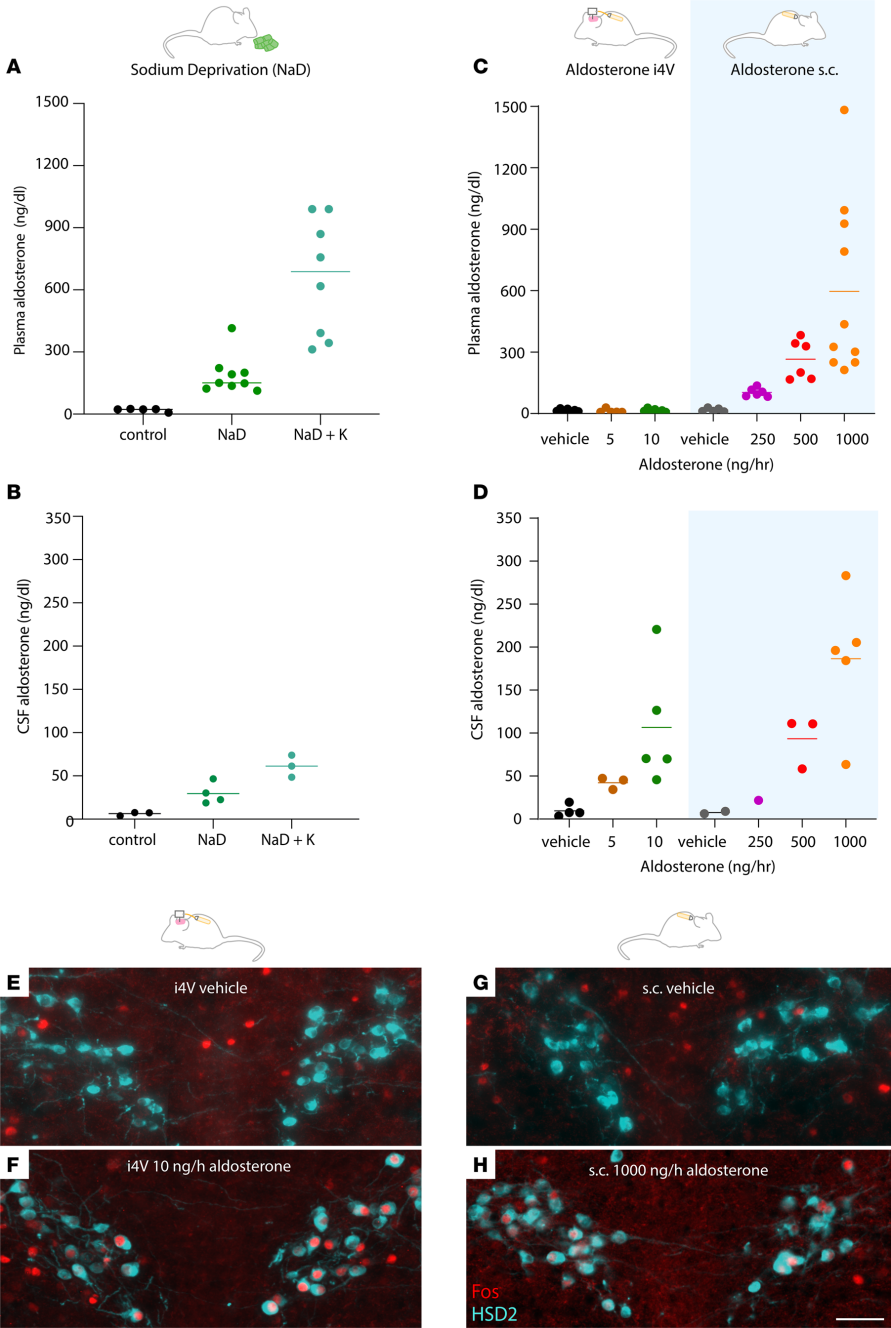
Effect of diet or aldosterone infusion on aldosterone concentration of blood plasma and cerebrospinal fluid (CSF). (**A**) Plasma aldosterone after dietary sodium deprivation (NaD) or NaD plus potassium supplementation (NaD+K). (**B**) CSF aldosterone after NaD or NaD+K. Each dot represents pooled CSF from 2–3 mice. (**C**) Plasma aldosterone in mice receiving aldosterone i4V (5–10 ng/h) or s.c. (250–1000 ng/h). (**D**) CSF aldosterone in mice receiving i4V or s.c. infusion. Each dot represents pooled CSF from 2–3 mice. For each group with more than 1 sample, the mean is represented by a horizontal line. (**E**–**H**) Aldosterone i4V and s.c. infusion induced Fos immunoreactivity in HSD2 neurons, while vehicle infusion did not. Scale bar: 50 μm.

The extent to which aldosterone crosses the blood-brain barrier remains unclear (43, 44), so we next considered the possibility that aldosterone concentrations in mice receiving i4V infusions were supraphysiologic. To address this possibility, we first measured endogenous aldosterone in blood and cerebrospinal fluid (CSF) across a variety of conditions, and we then assessed whether i4V infusions produced concentrations within or outside this range. We also tested whether peripheral infusions increased CSF aldosterone. Removing dietary sodium increased aldosterone in blood plasma to 113–414 ng/dL (Figure 5A) and elevated the CSF concentration to 19–47 ng/dL (Figure 5B). To determine the high end of endogenous production in mice, we adapted a human protocol that combines dietary sodium deprivation with potassium supplementation followed by acute potassium infusion (40), and we found higher aldosterone concentrations in blood plasma (313–990 ng/dL; Figure 5A) and CSF (48–74 ng/dL; Figure 5B).

Having determined the physiologic ranges of endogenous aldosterone in blood and CSF, we next examined the ranges produced by exogenous aldosterone infusion. As expected, i4V infusion increased the aldosterone concentration in CSF (Figure 5D), with no apparent change in blood plasma (Figure 5C). In contrast, peripheral aldosterone infusion increased both blood plasma and CSF aldosterone (Figure 5, C and D). Importantly, i4V infusion induced maximal salt intake at CSF aldosterone concentrations that overlap the range of endogenous CSF concentrations produced on a low-sodium diet. Specifically, CSF aldosterone concentrations were 34–47 ng/dL in mice with 5 ng/h i4V infusion (Figure 5D) and 19–47 ng/dL in sodium-deprived mice (Figure 5B). Altogether, our results indicate that circulating aldosterone reaches the brain and maximally increases salt intake at tissue concentrations of 34–47 ng/dL (0.9–1.3 nM), closely resembling its functional range of approximately 0.1–1 nM for binding and activating MR (45–48).

### Aldosterone activates HSD2 neurons.

Like other stimuli that increased saline intake (Figure 2), infusing aldosterone induced Fos expression in HSD2 neurons. We found robust Fos activation of HSD2 neurons in mice receiving central (i4V; Figure 5, E and F) and peripheral (s.c.; Figure 5, G and H) infusion.

### HSD2 neurons are required for aldosterone-induced saline intake.

**Figure 6:**
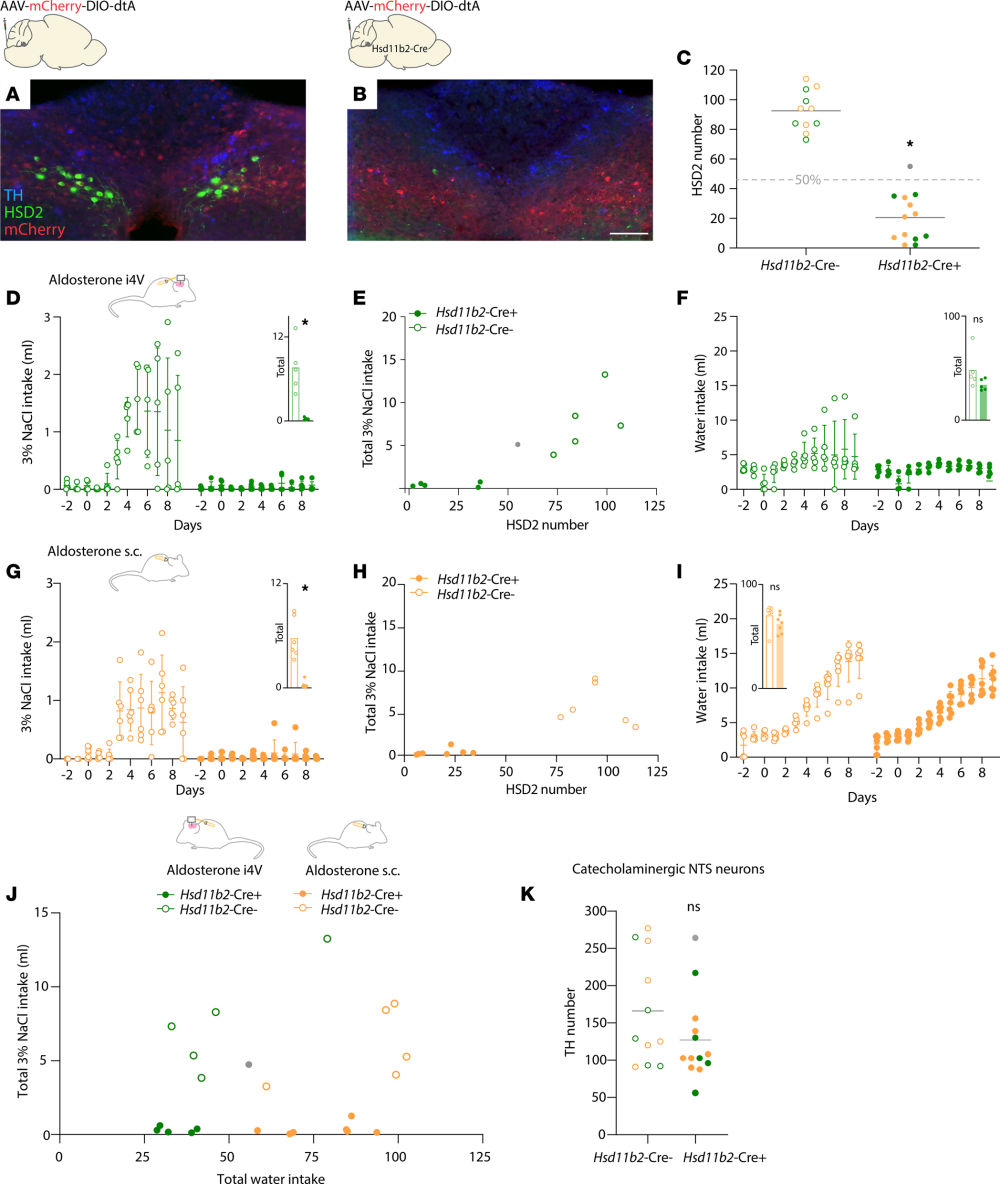
Effect of HSD2 neuron ablation on aldosterone-induced salt intake. (**A**) Intact distribution of 11-β-hydroxysteroid dehydrogenase-expressing neurons (HSD2, green) after bilateral injections of AAV-mCherry-DIO-dtA into the NTS of a Cre^–^ control mouse. (**B**) Few HSD2 neurons remained in an *Hsd11b2*-Cre^+^ experimental mice. Scale bar: 100 μm. In both **A** and **B**, neighboring catecholaminergic neurons are evident by their immunoreactivity for tyrosine hydroxylase (TH, blue). (**C**) This ablation method successfully reduced the number of HSD2 neurons (*P* < 0.0001). Gray dot indicates experimental case #3383, which we excluded from subsequent analysis because it did not meet the prespecified criterion of fewer than 50% of the average HSD2 neuron number in control mice. (**D**–**F**) Effects of HSD2 neuronal ablation on 3% NaCl and water intake in mice receiving i4V aldosterone (10 ng/h). Gray dot in **E** again represents case #3383. In **D**, inset, *P* = 0.0034. In **F**, inset, *P* = 0.1368. (**G**–**I**) Effects of HSD2 neuron ablation on 3% NaCl and water intake in mice receiving s.c. aldosterone (1,000 ng/h). In **G**, inset, *P* < 0.0001. In **I**, inset, *P* = 0.2106. (**J**) Scatter plot showing saline (*y* axis) versus water (*x* axis) intake of every experimental and control mouse receiving i4V or s.c. aldosterone. (**K**) *Hsd11b2*-Cre–dependent ablation did not alter the number of neurons NTS catecholaminergic (TH-immunoreactive) neurons (*P* = 0.1584). *P < 0.05 by unpaired, 2-tailed *t* test.

To test the hypothesis that HSD2 neurons are necessary for aldosterone-induced salt intake, we infused aldosterone at maximally effective doses i4V (10 ng/h) and s.c. (1000 ng/h). In experimental mice, we eliminated HSD2 neurons by injecting Cre-dependent diphtheria toxin A (AAV1-mCherry-DIO-dtA) bilaterally into the NTS of *Hsd11b2*-Cre mice (Figure 6B). In control mice, we injected the same Cre-dependent vector into the NTS of Cre^–^ littermates (Figure 6A). This approach substantially reduced the number of HSD2 neurons (*P* < 0.0001 relative to Cre^–^ littermates; Figure 6C) and eliminated saline intake induced by i4V aldosterone infusion (*P* = 0.0034 relative to Cre^–^ littermates; Figure 6, D–F) and by peripheral aldosterone infusion (*P* < 0.0001 relative to Cre^–^ littermates; Figure 6, G and H). In contrast, ablating HSD2 neurons did not reduce water intake caused by peripheral aldosterone infusion (Figure 6, I and J).

Experimental mice with up to 36 (i4V) or 34 (s.c.) remaining HSD2 neurons consumed little to no saline, and examining the relationship between saline intake and HSD2 neuron number (Figure 6, E and H) suggested that aldosterone-induced sodium appetite can be prevented by eliminating 50% of the HSD2 neuron population. One *Hsd11b2*-Cre^+^ mouse failed our inclusion criterion of > 50% ablation (55 surviving HSD2 neurons; Figure 6, C and E), and this mouse was the only member of the experimental group with salt intake in the control range.

**Figure 7:**
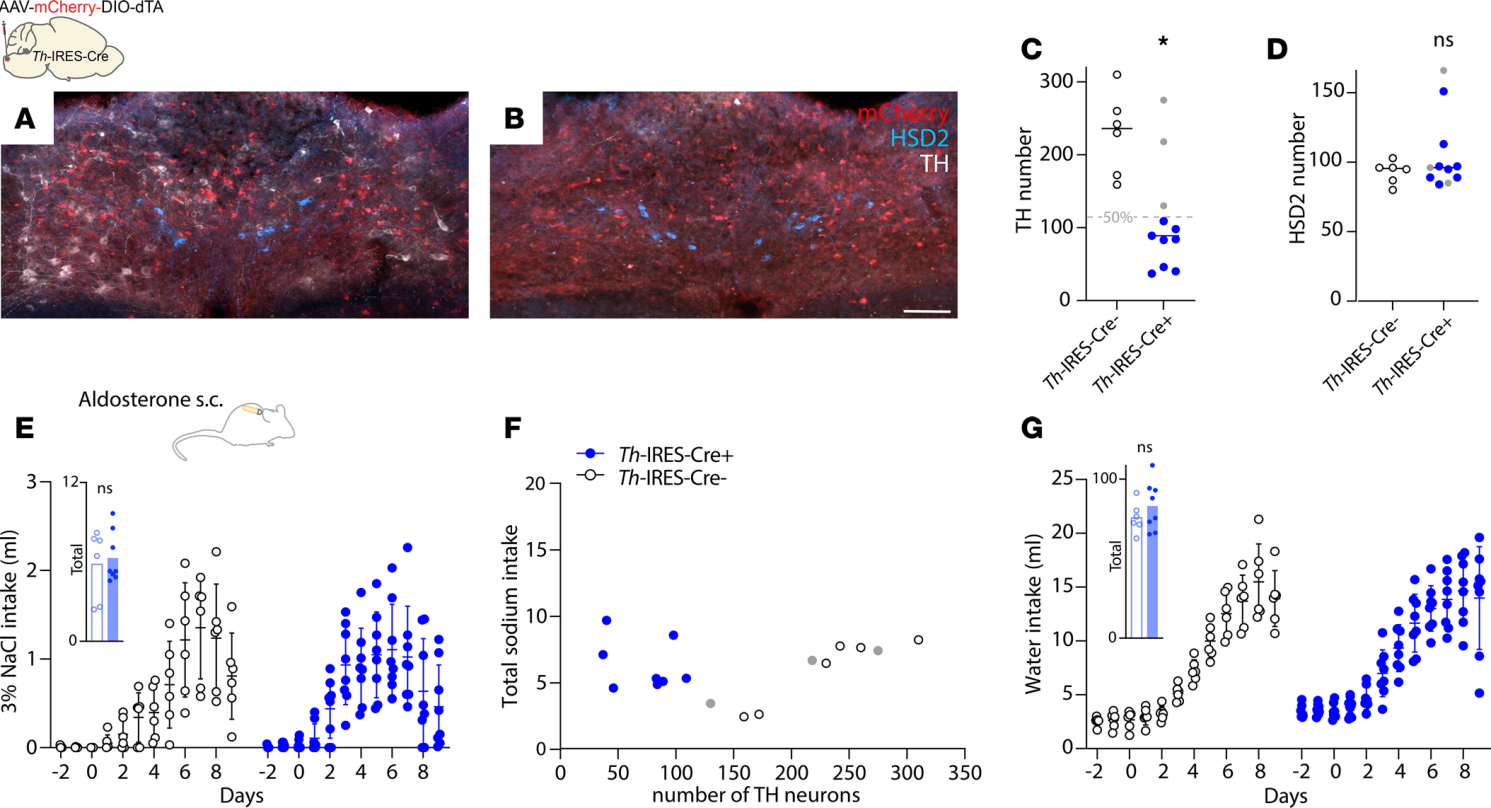
Effect of catecholaminergic neuron ablation on aldosterone-induced salt intake. (**A**) Intact distribution of TH-immunoreactive neurons (white) after bilateral injections of AAV-mCherry-DIO-dtA into the NTS of a Cre^–^ control mouse. (**B**) Few TH^+^ neurons remained in *Th*-IRES-Cre^+^ experimental mice. Scale bar: 100 μm. In both **A** and **B**, HSD2 neurons (blue) are unaffected. (**C**) Genetically targeted ablation reduced the number of catecholaminergic neurons (*P = 0.04 by 2-tailed *t* test, relative to Cre^–^ littermates) without altering the number of HSD2 neurons (*P* = 0.2886). (**E**–**G**) Effects of catecholaminergic neuron ablation on 3% NaCl and water intake in mice receiving s.c. aldosterone infusion (1,000 ng/h). Insets in **E** and **G** show *P* = 0.7120 and *P* = 0.3315, respectively, by 2-tailed *t* test versus Cre^–^ littermates.

To assess ablation specificity, we also counted the neighboring population of catecholaminergic neurons, which are immunoreactive for the enzyme tyrosine hydroxylase (TH). Cre-dependent ablations in *Hsd11b2*-Cre mice did not alter the number of NTS catecholaminergic neurons (*P* = 0.1584 relative to Cre^–^ littermates; Figure 6K). We also tested whether aldosterone-induced sodium appetite depends exclusively on HSD2 neurons by using a similar approach to ablate NTS catecholaminergic neurons in *Th*-IRES-Cre mice (Figure 7, A and B). This approach eliminated TH-immunoreactive neurons in *Th*-IRES-Cre^+^ experimental mice (*P* = 0.04 relative to Cre^–^ littermates; Figure 7C), without altering the number of HSD2 neurons (*P* = 0.2886; Figure 7D) and without reducing aldosterone-induced saline intake (*P* = 0.7120; Figure 7E). Saline consumption had no apparent relationship to the number of surviving catecholaminergic neurons (Figure 7F), and all mice drank more water during s.c. aldosterone infusion, with no difference between groups (*P* = 0.3315; Figure 7G). Thus, aldosterone-induced sodium appetite requires HSD2 neurons and not the larger, neighboring population of catecholaminergic neurons in the NTS.

Aldosterone increases HSD2 expression, which improves visibility of HSD2-immunolabeled cells (31). We took advantage of this and updated our previous estimate of the total number of HSD2 neurons in mice by combining neuron counts from mice in *Th*-IRES-Cre experimental and control groups with both groups of Cre^–^ mice from the HSD2 ablation experiment (*n* = 28 total). Multiplying the number of HSD2 neurons in each 1-in-3 tissue series (97.7 ± 20.1) by 3 and then applying the Abercrombie correction (49) with an average nuclear diameter of 9.1 ± 0.1 μm (28) produced an estimated 239 ± 16 HSD2 neurons per mouse. This number is double our original estimate in mice (28) and one-fourth the number of HSD2 neurons in humans.

### Water intake during peripheral infusion of aldosterone.

We did not expect the increase in water intake during peripheral (s.c.) infusion of aldosterone, and the lack of an effect with i4V aldosterone, plus the persistence of this effect in mice lacking HSD2 neurons, suggested a peripheral mechanism. Several factors can increase thirst, and it is important to first differentiate “primary” polydipsia, thought to have a neurologic or psychiatric cause, from thirst triggered by excessive urinary loss, so we began by comparing daily water intake and urine output between aldosterone- and vehicle-infused mice with and without mildly restricted access to water. These mice had ad libitum access to normal chow and no access to saline. With unrestricted access, aldosterone-infused mice drank more water (*P* < 0.0001 relative to vehicle-infused controls), with intakes gradually rising above 10 mL per day. These mice excreted proportionally more urine (*P* = 0.0055; Supplemental Figure 2, D–F), with no differences in body weight change (aldosterone: –1.2 ± 0.6 g vs. vehicle: –0.2 ± 0.7 g, *P* = 0.0769) or total food intake (aldosterone: 39.1 ± 2.6 g vs. vehicle: 36.3 ± 2.0 g, *P* = 0.1459). However, with mildly restricted access to water (5 mL per day), daily urine volumes of aldosterone-infused mice remained within the range of vehicle-infused mice (aldosterone vs. vehicle d1–d7, *P* > 0.9999). These water-restricted, aldosterone-infused mice did not lose weight (*P* = 0.3268), and their daily urine output did not increase until we liberalized their access to water (aldosterone d1–d7 vs. d8–d10; *P* = 0.0071; aldosterone d8–d10 vs vehicle d8–d10, *P* = 0.0010; Supplemental Figures 2, G–I). Diabetes insipidus is a well-known cause of polyuria and polydipsia, and in separate groups of aldosterone- and vehicle-infused mice, we measured plasma copeptin, which is produced in direct proportion to vasopressin (the antidiuretic hormone), but there was no difference between groups (*P* = 0.0907; Supplemental Figure 2L). Hyperglycemia is another well-known cause of polyuria and polydipsia, and aldosteronism is associated with elevated risk of diabetes mellitus (7, 9), but blood glucose levels were not elevated in aldosterone-infused mice (*P* = 0.1571; Supplemental Figure 2M). We also considered the possibility that aldosterone-infused mice were thirsty due to hypernatremia, but the plasma sodium concentration was not elevated in aldosterone-infused mice (*P* = 0.9523; Supplemental Figure 2K). Thus, polydipsia evoked by peripheral aldosterone infusion does not result from hypernatremia, hyperglycemia, diabetes insipidus, or polyuria.

## Discussion

This study improves our understanding of sodium appetite by clarifying the dose-response relationship between aldosterone and salt intake and identifying the neurons required for this behavioral effect. We showed that dietary sodium deprivation elevates aldosterone, which crosses the blood-brain barrier at concentrations within its functional range for activating MR. We also showed that aldosterone activates HSD2 neurons, confirmed that activating HSD2 neurons increases sodium appetite, and discovered that eliminating HSD2 neurons prevents aldosterone-induced salt intake. Additionally, we identified homologous neurons in humans and pigs, suggesting that these aldosterone-sensitive, sodium appetite–promoting neurons are an evolutionarily conserved component of the mammalian brain. Collectively, these findings highlight HSD2 neurons as a target for future therapies that modify human sodium intake.

### Aldosterone increases salt intake.

Our findings unequivocally demonstrate that aldosterone increases sodium appetite in mice. We found specificity for saline intake, a clear dose-response relationship, and neuroanatomical localization of the effect, with high i4V potency relative to forebrain or peripheral delivery. This behavioral effect complements aldosterone’s sodium-retaining effect in peripheral epithelia, making it a key regulator of extracellular fluid volume.

These findings update previous misconceptions, including our own, about the likely relevance of mineralocorticoid-induced behavior. Knowing that aldosterone poorly penetrates the blood-brain barrier (44, 47, 50) raised doubts as to whether it reaches tissue concentrations adequate to influence behavior (43). Although a different MR agonist, deoxycorticosterone, increased salt intake in rats (51, 52), this required supraphysiologic doses, and subsequent investigators could not replicate the effect in mice (36, 53), raising doubt about the likelihood that aldosterone plays a behavioral role.

In contrast, our dose-ranging tests revealed that a modest but meaningful portion of circulating aldosterone penetrates the blood-brain barrier. One week of a low-sodium diet triggered enough aldosterone production to elevate brain (CSF) concentrations into the low-nanomolar range, in which it binds and activates the MR. Importantly, peak salt intake occurred at i4V infusion rates reproducing precisely the range of CSF concentrations we measured in salt-hungry mice after a week of dietary sodium deprivation. These findings, along with a previous finding that i4V injection of an MR antagonist inhibits saline intake in sodium-deprived rats (11), indicate that the large rise in circulating aldosterone caused by dietary sodium deprivation contributes to sodium appetite.

Dietary sodium deprivation with potassium supplementation is an ethologically relevant paradigm for triggering aldosterone production (1, 2, 32, 54) and sodium appetite (32, 55, 56). These 2 dietary manipulations recreate the dilemma faced by land-dwelling herbivores and omnivores; most foods have high potassium and zero sodium. While a high-potassium diet is beneficial, a zero-sodium diet is harmful and eventually lethal (57). Excreting potassium on an low-sodium diet requires increased aldosterone (40, 58), and the chronic hypovolemia that results from inadequate dietary sodium further elevates aldosterone (1, 32, 59).

Our results confirm that, like rats and humans, mice generate very high aldosterone levels (100–1,000 ng/dL) within a week of altering dietary sodium and potassium. Prior to this study, it was unclear why the body expends so much energy to undergo hypertrophy in the adrenal glomerulosa and generate such high levels of circulating aldosterone (10–20 nM). These levels are well above what is needed to activate MR in peripheral HSD2-expressing cells, but based on the results of this study, we propose that the benefit of generating such high levels of circulating aldosterone is that these levels overcome a steep concentration gradient between the blood and the brain (44). Blood levels at or below 50–100 ng/dL (1–3 nM) are more than sufficient to activate MR in HSD2-expressing epithelial cells in the kidney, but activating the behavioral drive to seek and consume sodium requires higher circulating levels (well above 100 ng/dL) to activate MR in HSD2 neurons. Mild elevations are adequate for sodium retention, but larger amounts of aldosterone are needed in a chronic zero-sodium environment to achieve brain concentrations adequate for activating the behavioral mode that represents the only way to repair a chronic volume deficit: seeking and consuming salt.

Like mice and rats, humans can generate circulating aldosterone levels upward of 300 mg/dL on a low-sodium, high-potassium diet (40). Even higher levels have been found in patients with aldosterone-secreting adenomas or chronic kidney disease (41, 42). Besides individuals deliberately abstaining from sodium (60, 61) and some indigenous tribes (59), most people consume more than enough sodium and probably will never generate such high aldosterone levels. Thus, it is important to determine the extent to which aldosterone induces sodium appetite in healthy, sodium-deprived patients, but it is even more important to investigate its effect in patients with aldosteronism.

### Cell-type specificity of aldosterone-induced salt intake.

Several previous investigators attempted to localize the site of action for mineralocorticoid-induced sodium appetite in the rat brain. In the 1960s, Wolf and colleagues found that electrolytic lesions in the hypothalamus reduced several ingestive behaviors, including deoxycorticosterone-induced saline intake (62). In the 1980s, Epstein and colleagues proposed that sodium appetite arises from synergy between aldosterone and angiotensin II (63), acting in or near the anterior wall of the third ventricle (64). In the present study, infusing aldosterone near this region did not alter salt intake, similar to a previous result in rats (11). In yet another forebrain region, the central nucleus of the amygdala, mineralocorticosteroid-induced saline intake was reduced by injecting either excitotoxins or antisense oligonucleotides designed to suppress MR production (65–68). However, aldosterone is unlikely to act directly within any of these regions because no cells in the forebrain express both HSD2 and MR (28, 37), although the central nucleus of the amygdala does contain neurons with direct output projections to the HSD2 neurons in the NTS (69).

While angiotensin II potently increases water intake (70), aldosterone is the most selective and potent stimulus for salt intake. Infusing aldosterone suppresses angiotensin II (71), and the present results confirm that it is capable of inducing salt intake. This singular ability of aldosterone, coupled with coexpression of MR and HSD2 in a singular population of neurons, provided circumstantial evidence for involvement of these neurons in aldosterone-induced salt intake. Further circumstantial support derived from the finding that HSD2 neurons fire faster after chronic infusion of aldosterone (33) and trigger saline intake when chemogenetically stimulated (33, 34). Despite this compelling circumstantial evidence, directly testing this hypothesis required, first, determining an effective dose of aldosterone in mice and then selectively removing HSD2 neurons to assess their role. This combined approach unambiguously showed that HSD2 neurons are necessary for aldosterone-induced sodium appetite. They are the first and, thus far, only neurons with this property.

Boosting sodium appetite may be the only central function of HSD2 neurons, as neither i4V infusion of aldosterone nor chemogenetic activation of HSD2 neurons altered blood pressure (12, 33). Thus, one hormone, aldosterone, induces a highly specific behavioral program, seeking and consuming sodium, and this behavioral switch depends upon a population of roughly 200 neurons in mice and 1,000 neurons in humans. HSD2 neurons are a minority of neurons within the NTS and an even smaller fraction of neurons in the brain — 0.0003% of the 71 million neurons in the mouse brain (72) and 0.000001% of 86 billion neurons in the human brain (73). We are not aware of any other example of a mammalian behavior depending so completely on the integrity of so few neurons. The total dependency of aldosterone-induced salt intake on the integrity of HSD2 neurons may represent the most delicately cell-type–specific behavioral dependency in the mammalian brain.

### Clinical relevance.

Our findings have important implications for patients in whom dietary sodium is known to modify symptom severity or end-organ damage. Our finding of HSD2 neurons in the human brainstem suggests translational potential, and the existence of neurons that promote sodium appetite provides an opportunity for targeted interventions that modify sodium consumption and its associated risks and benefits in vulnerable patients.

Specifically, the HSD2 neurons may be a useful lever for nudging sodium intake up or down, depending on the need in a specific patient. For example, salt supplementation is helpful in many patients with orthostatic hypotension and vasovagal syncope caused by autonomic disorders like postural orthostatic tachycardia syndrome or by neurodegenerative synucleinopathies like Parkinson’s disease, dementia with Lewy bodies, and multiple system atrophy (60, 74, 75). Dietary sodium is often supplemented with large salt tablets in combination with a mineralocorticoid-glucocorticoid receptor agonist, fludrocortisone, but salt tablets cause gastrointestinal distress and fludrocortisone is contraindicated in patients with supine hypertension, which is common in autonomic synucleinopathies (75). In such patients, graded stimulation of HSD2 neurons could boost intrinsic drive to consume salty foods. The efficacy of stimulating HSD2 neurons in mice confirms the basic feasibility of this approach.

Separately, ablating or inhibiting HSD2 neurons may help people with salt-sensitive hypertension (76), particularly those with aldosteronism, which is a common cause of treatment-refractory hypertension (17–22). Patients with aldosteronism suffer higher rates of stroke, heart failure, and other cardiovascular complications, and their increased sodium intake explains much of this elevated risk (17–20). Cutting salt intake improved blood pressure in a 12-week trial (22), but we lack efficacious therapies for sustaining this dietary change. Our cell-type–specific ablation result establishes the feasibility of eliminating HSD2 neurons in mice, but additional work is required to establish feasibility and then evaluate efficacy and safety in patients.

Overall, an important implication of our results is that dietary sodium is a regulated variable. The behavioral motivation to consume salt, much like the motivation to consume food or water, is subject to homeostatic feedback and governed by neurons in the brain. Patients and the general public stand to benefit if we incorporate this information into nutritional recommendations and public health policies involving the choices people make about what they eat and drink.

### New questions and limitations.

Our results included unexpected findings that represent potentially new opportunities for investigation, and several limitations inherent to our approach warrant discussion. First, despite identifying homologous neurons in the human brain, our reliance on an animal model necessitates cautious extrapolation to human behavior. Further investigation in humans is warranted.

Second, peripheral infusion induced less salt intake than i4V infusion, even at doses that generated CSF aldosterone levels above the range of maximally effective i4V infusions, so this difference cannot be explained by inadequate aldosterone in the brain. Salt consumption increases plasma sodium concentration and volume (32), both of which reduce sodium appetite (26, 31, 32, 77–79), but none of our aldosterone-infused mice were hypernatremic, and i4V-infused animals probably excreted all of the excess saline they consume (12). In contrast, the high circulating aldosterone levels of peripherally infused mice likely caused sodium retention and blood volume expansion, which activate low-pressure barosensors, trigger cardiac release of natriuretic peptides, and suppress angiotensin II production. It will be helpful to clarify the effect of these neuroendocrine mechanisms on HSD2 and other neurons in the overall network controlling salt-ingestive behavior.

Third, the mechanism of increased water intake caused by peripheral aldosterone infusion is unclear. Aldosterone-induced thirst, which also occurs in rats (80), does not appear to involve well-known thirst triggers like hypernatremia, hyperglycemia, diabetes insipidus, polyuria, or hypovolemia. Similar to salt, daily water intake increased gradually and then plateaued over the course of a week. In contrast to salt, stimulating HSD2 neurons did not increase water intake, and eliminating HSD2 neurons did not reduce peripheral-aldosterone–induced thirst. Also, thirst appeared at peripheral infusion rates below the threshold for salt intake, and infusing aldosterone into the cerebral ventricles had little to no effect on water intake, indicating that this effect does not involve direct action in the brain. It would be interesting to uncover the mechanism of this effect.

Fourth, salt intake induced by chronic chemogenetic stimulation of HSD2 neurons peaked by the first day of CNO infusion, while aldosterone induced a more gradual rise in salt intake, resembling the slow rise in saline intake following consecutive daily injections of deoxycorticosterone or i4V aldosterone infusion in rats (11, 12, 31, 51). This gradual time course, along with the lack of an acute effect of aldosterone on HSD2 neurons in ex vivo brain tissue slices (33), is more consistent with a gradual change in gene transcription evoked by MR than with previous suggestions of a rapid, nongenomic effect (68, 81). Further supporting involvement of this nuclear steroid receptor, aldosterone-induced salt intake was maximal at CSF concentrations of approximately 1 nM, which is the peak concentration for aldosterone binding and transcriptional activation of MR (45–48). It will be important to determine exactly how MR activation increases HSD2 neuronal activity.

And fifth, more investigation is needed to understand the full neural network that regulates sodium appetite. We proposed that HSD2 neurons integrate both synaptic and endocrine inputs, but the nature of this synaptic input and how exactly aldosterone and other endocrine signals modulate it remain unclear (33, 43). HSD2 neurons send output to 2 primary targets (28, 33, 34, 82). One of these, the fusiform subnucleus of the bed nucleus of the stria terminalis, may embody a network interaction between aldosterone-sensitive HSD2 neurons in the NTS and angiotensin-sensitive neurons in the lamina terminalis (83). Their other target, the prelocus coeruleus, may integrate input from the HSD2 neurons with information from the medial prefrontal cortex and from appetite-regulating neurons in the hypothalamus (82). Fully understanding this aldosterone-modulated network will require studying the connections and functions of each node.

### Conclusion.

A singular hormone (aldosterone) activates a specific behavior (salt intake) via distinct cells (HSD2 neurons). Identifying the cells necessary for aldosterone-induced salt intake improves our understanding of the neural circuitry controlling sodium appetite and provides a promising target for therapeutic strategies to boost sodium appetite in patients with hypovolemia and to mitigate excessive salt intake in patients with aldosteronism.

## Methods

### Sex as a biological variable.

Sex was not considered as a biological variable in these studies. Due to increased variability of sodium appetite in female rodents (56), we performed all experiments in male mice.

### Human, porcine, and rat brainstem tissue.

We obtained the caudal brainstem from 12 human brains removed after death from a variety of nonneurologic causes (Table 1). The postmortem interval between death and autopsy was 33.5 ± 18 hours (mean ± SD; range 15–50 hours). Average age at death was 35 ± 29.6 years (range: 33 gestational weeks to 77 years). Sex was female in 2 cases and male in 9. After fixing for 2–3 days at 4°C in 10% formalin-PBS (SF100-20, Thermo Fisher Scientific), we cryoprotected the brainstem in 30% sucrose-PBS for an additional 1–2 days until it sank. Next, we cut 40 μm–thick tissue sections in the transaxial plane using a freezing-stage microtome. We collected tissue in a separate 1-in-12 series with approximately 0.5 mm between successive sections in each series, which were stored in cryoprotectant at –30°C. We used the same protocol to process brainstem tissue, gifted to us from Michael Welsh at the University of Iowa, from 4 pigs that were euthanized and autopsied after previous research studies. For rat brainstem tissue, we obtained unpublished images from slides prepared and described in a previous publication (84).

### Mice.

All mice were group-housed in a temperature- and humidity-controlled room with a 12/12-hour light/dark cycle and, initially, ad libitum access to water and standard rodent chow (Envigo 7013). Some were provided low-sodium chow (TD-130591, Teklad/Envigo) for 1 or more days in protocols described below. In addition to C57BL/6J mice, we used hemizygous *Hsd11b2*-Cre or *Th*-IRES-Cre mice maintained on a C57BL/6J background (Table 2).

### Stereotaxic injections.

To selectively ablate Cre-expressing neurons in the NTS, we used a head-down stereotaxic approach (82, 85) to inject AAV-DIO-dtA-mCherry [AAV1-Ef1a-Lox-Cherry-lox(dtA)-lox2.ape, 5.2 × 10^12^ vg/mL; UNC Gene Therapy Center Vector Core]. After post hoc histology (described below), we included all experimental-ablation mice for analysis if they had fewer than 50% of the average number of HSD2 neurons counted control mice. To chemogenetically stimulate HSD2 neurons, we instead injected AAV-DIO-hM3Dq-mCherry (AAV1-Ef1a-DIO-hM3Dq-mCherry, 4 × 10^12^ vg/mL; Duke Viral Vector Core). In additional mice with CNO continuous infusion pumps, we used AAV8-DIO-hM3Dq-mCherry (44361-AAV8, Addgene; 2.1 × 10^13^ gc/mL), and control mice received AAV8-DIO-mCherry (50459-AAV8, Addgene; 2.2 × 10^13^ gc/mL).

### Cannula implantation and s.c. osmotic minipumps.

Under anesthesia, the scalp was shaved, and the mouse was placed gently in the stereotaxic ear bars. A scalp incision was made wide enough to expose the skull at both bregma and lambda. After leveling the skull (both AP and ML) and zeroing at bregma, a 1 mm burr hole was made using a high-speed rotary tool with a 0.8 mm bit. An L-shaped 28-gauge cannula (3280PM-SPC, 5 mm; Plastics One) was attached by tubing (0.69 mm ID × 1.14 mm OD; Scientific Commodities) to an osmotic minipump (ALZET 1007D) then inserted through the burr hole to the target coordinate (i4V: 0 mm lateral, –6.5 mm caudal, and 5.0 mm deep to bregma for i4V; lateral ventricle [LV]: 0.8 mm right, 0.35 mm caudal, and 2.5 mm deep to bregma). Cyanoacrylate glue with dental cement was used to fix the cannula. After the cannula was firmly cemented in place, the minipump was inserted into a s.c. pocket in the midscapular area. To infuse aldosterone peripherally, we placed the osmotic minipump in the same s.c. position without connecting any cannula or tubing. The skin was closed using Vetbond (3M). In every mouse with an i4V or LV cannula, under anesthesia and immediately before transcardial perfusion, we disconnected the catheter from the minipump and used a 28 gauge syringe to inject Evans blue dye (1% in sterile water) to check cannula patency. We excluded from analysis all mice without blue dye in the i4V or LV.

### BioDAQ fluid intake measurement.

Mice were housed individually in BioDAQ cages (Research Diets) as described previously (39). Each cage had fresh ALPHA-dri+ Plus bedding (Shepherd Specialty Papers), an overhead hopper with standard rodent chow (Teklad 7013), and 2 bottles (distilled water and 3% NaCl) that could be blocked by a gate. Fluid intake was recorded continuously, but we analyzed data from prespecified intervals, as detailed below.

### Dietary sodium deprivation and potassium supplementation.

We habituated mice in individual BioDAQ cages with low-sodium chow and ad libitum access to water and 3% NaCl for at least 3 days. We then closed the access gate for 3% NaCl within 30 minutes before lights-off. To prevent consumption of excreted sodium, the mouse was provided with a fresh, clean cage every day. Six days later, we reopened the saline access gate 10 minutes before lights-off and measured 3% NaCl and water intakes across the following 6 hours.

For dietary sodium deprivation plus potassium supplementation, we modeled our approach after a similar dietary protocol followed by acute infusion of potassium chloride (KCl) in patients (40). However, instead of i.v. infusion at the end of our protocol, we gave a bolus of potassium by gavage (3M KCl, 0.1 mL per 10 g), similar to previous rodent experiments (86). Also, after pilot experiments testing a range of concentrations, we found that mice show no aversion to 1.5% or less KCl in drinking water (they consumed this solution at the same daily volumes as dH_2_O). Thus, our protocol for dietary sodium deprivation plus potassium supplementation was ad libitum access to 1.5% KCl in the drinking water, plus the same low-sodium chow as above, and fresh cage changes. After 6 days, these mice (*n* = 8) were perfused immediately following CSF and blood collection.

### Aldosterone dose-response testing.

We infused aldosterone using ALZET 1007D osmotic minipumps, which deliver 0.5 μL/h. The day before implanting, we preloaded each minipump with 100 μL of vehicle (1% ethanol in sterile 0.9% saline) or aldosterone solution and then placed it in sterile saline overnight. We tested separate doses in separate groups of mice, with each mouse receiving 1 dose.

For i4V infusions, we monitored saline and water intake for up to 10 days in separate groups receiving either vehicle (*n* = 4) or aldosterone 1 (*n* = 3), 2.5 (*n* = 2), 5 (*n* = 6), 10 (*n* = 5), 25 (*n* = 5), 50 (*n* = 4), or 100 (*n* = 5) ng/h. After identifying a peak effect at 10 ng/h aldosterone (i4V), we used an additional group of mice to infuse that dose into the lateral ventricle (*n* = 7). We discarded 3 days of data (2 presurgery, 1 day-of surgery) from 1 mouse (1 ng/h i4V aldosterone group) with aberrant readings caused by a loose fluid hopper.

For peripheral infusions, we monitored saline and water intake for up to 10 days in separate groups receiving either vehicle (*n* = 4) or aldosterone 10 (*n* = 2), 100 (*n* = 3), 250 (*n* = 3), 500 (*n* = 5), 750 (*n* = 3), or 1,000 (*n* = 6) ng/h.

### Cell-type–specific neuronal stimulation and ablation.

Four weeks after injecting AAV-DIO-hM3Dq-mCherry into the NTS of *Hsd11b2-*Cre mice, and after at least 3 days of habituation to individual BioDAQ cages, we injected mice 30 minutes prior to lights-off with either CNO (1 mg/kg in sterile 0.9% NaCl with 1% DMSO) or sterile 0.9 % NaCl (0.1 mL per 10 g mouse). After at least 3 additional days, we repeated the injection with the other solution (vehicle or CNO). We analyzed water and 3% NaCl intake across the 6 hours after lights-off. In additional cohorts, we habituated mice to individual BioDAQ cages for 5 days and then implanted an ALZET 1007D minipump (delivering CNO 0.5 mg/kg/h s.c.) and continued monitoring water and 3% NaCl intake for 7d. At the end of each experiment, we perfused each mouse for histological assessment of HSD2 neuron transduction.

In separate cohorts, we stereotaxically microinjected AAV-DIO-dtA-mCherry in *Hsd11b2-*Cre experimental mice (*n* = 19 i4V aldosterone; *n* = 12 s.c. aldosterone) and in Cre^–^ littermates as controls (*n* = 10 i4V aldosterone; *n* = 6 s.c. aldosterone). Four weeks later, mice were habituated in BioDAQ cages for at least 3 days. We then implanted osmotic minipumps, returned each mouse to its BioDAQ cage, and monitored fluid intake for 9 more days. We repeated this protocol with s.c. aldosterone infusion in *Th*-IRES-Cre (*n* = 8) and Cre^–^ littermate control mice (*n* = 11).

### Metabolic cage studies.

To test whether peripheral aldosterone infusion increased urinary fluid loss, we used 4 groups of mice. Two received aldosterone (1,000 ng/h s.c.) and 2 received vehicle. Mice were habituated for 3d in individual metabolic cages with ad libitum food and water access (Ugo Basile, 41700). Urine was collected, and water and food intake were measured daily. After s.c. minipump implantation, mice were returned to the metabolic cages for 10d. Two groups (aldosterone and vehicle, *n* = 4 each) had ad libitum water access, and the other 2 (aldosterone and vehicle, *n* = 4 each) received 5 mL water — which is slightly more than vehicle-infused mice drank but less than s.c. aldosterone-infused mice drank in our initial BioDAQ recordings — every day for 7d; they then gained ad libitum water access for the final 3d. In addition to expected evaporative losses, 2–3 urine samples from 2 mice in the 5 mL water–restricted experiment had fecal contamination, requiring removal of 2 data points from animal 7863 (days 6 and 10) and 3 data points from animal 7864 (days 1, 5, and 6).

### Measurements in blood plasma and CSF.

After 6 days of aldosterone infusion, or dietary sodium deprivation with or without potassium supplementation, we collected blood and CSF. Prior to collection, mice receiving s.c. infusion (vehicle and 250, 500, and 1,000 ng/h aldosterone) were housed in standard cages with ad libitum access to regular chow and water. Mice receiving i4V infusion (vehicle and 5 and 10 ng/h aldosterone) were monitored in BioDAQ cages with ad libitum access to regular chow, dH_2_O, and 3% NaCl. Because CSF collection precluded our blue-dye assay for i4V cannula patency, we used a behavioral assay for cannula patency and only collected blood and CSF from mice that were consuming at least 1 mL of 3% NaCl per day by d6, which was at the low end of our dose-response results with 5 and 10 ng/h i4V aldosterone infusion (above). Mice consuming little to no 3% NaCl were presumed to have a nonpatent cannula and euthanized without blood or CSF collection.

To collect CSF, we used a pulled glass capillary pipette (80–100 μm tip inner diameter). We anesthetized each mouse and, using the NTS surgical exposure described above, inserted the tip slowly through the atlantooccipital membrane until CSF flowed spontaneously from the cisterna magna into the pipette, which was left in place for 10–30 minutes. CSF volumes collected from most mice ranged from 3 to 12 μL. After slowly removing the pipette, CSF was ejected from the pipette into a 200 μL tube by inserting a 22 gauge blunt needle and using an attached 10 mL syringe to push air into the pipette. CSF was stored at –80°C.

Immediately after collecting CSF, we injected ketamine/xylazine for terminal blood collection and euthanasia. After 5 minutes, we opened the peritoneum and collected blood from the inferior vena cava with an insulin syringe/needle. Blood was ejected from the insulin syringe into a microcentrifuge tube before being spun for 5 minutes at 6,000*g* at 4°C. Serum was extracted using a pipettor and stored at –80°C.

We used an ELISA to measure the aldosterone concentration (IB79134, IBL) in mouse blood plasma and pooled CSF. After thawing and diluting samples (1:10 for aldosterone), we followed the standard instructions for each commercially available sandwich ELISA kit. In a subset of mice receiving s.c. infusion of aldosterone (1,000 ng/h) or vehicle, we used an ion-sensitive electrode to measure serum sodium (Prolyte; Diamond Diagnostics). To measure plasma osmolality, we also used an osmometer. In an additional cohort of mice receiving s.c. infusion of aldosterone (1,000 ng/h) or vehicle, we provided ad libitum access to food and water (no saline), for 7d; then, we collected blood from the mandibular vein and centrifuged as above, extracted, and stored serum at –80°C. In these samples, we measured copeptin with an ELISA kit (MBS160428, MyBioSource) used in previous work in mice (87). We also measured blood glucose using a glucometer (FreeStyle Lite, Abbott).

### Transcardial perfusion and tissue sectioning.

All mice with stereotaxic AAV injection or implanted cannulas and a subset of mice with s.c. infusion pumps were transcardially perfused as follows. First, each mouse was anesthetized with ketamine (150 mg/kg) and xylazine (15 mg/kg), dissolved in sterile 0.9% saline, and injected i.p. It was then perfused transcardially with phosphate-buffered saline (PBS) prepared from 10× stock (P7059, MilliporeSigma), followed by 10% formalin-PBS (SF100-20, Thermo Fisher Scientific). After perfusion, we removed each brain and fixed it overnight in 10% formalin-PBS. We sectioned each brain into 40 μm–thick coronal slices using a freezing microtome and collected tissue sections into separate, 1-in-3 series. We stored all tissue sections in cryoprotectant solution at –30°C.

### Immunolabeling and in situ hybridization.

To label HSD2 in human and porcine brain tissue sections, we used a rabbit polyclonal antiserum (Table 3) and nickel-diaminobenzene (NiDAB) IHC as described in previous work (28). For human cases, we selected 16–20 total sections from one 1-in-12 series spanning approximately 1 cm of the caudal medulla oblongata. This tissue series contained intermediate through caudal levels of the human NTS, as well as the cuneate, gracile, and spinal trigeminal nuclei, plus the caudal inferior olivary complex and pyramidal tracts back through the spinomedullary transition. For immunofluorescence labeling, we used previously described protocols (28, 39, 88) and primary antisera in Table 3. To label mRNA for *HSD11B2*, we selected adjacent tissue sections from 5 human cases with immunolabeling for HSD2. We then used the RNAscope 2.5 HD Detection Reagent-BROWN (322310; Advanced Cell Diagnostics [ACD]) and a probe for human *HSD11B2* mRNA (432351; ACD) with a previously described protocol (39).

### Imaging, cell counts, and figures.

All slides were scanned using a VS120 microscope and VS-ASW software (Olympus). We acquired a 2× overview and 10× whole-slide images, followed by 20× and, in some cases, 40× *Z* stacks encompassing the NTS at every level containing HSD2 neurons. We reviewed slides in OlyVIA (Olympus) and then used cellSens (Olympus) to crop full-resolution images and Adobe Photoshop to adjust brightness and contrast. We used Adobe Illustrator to arrange panels and add lettering for figure layouts.

For bright-field analysis in human tissue, we identified HSD2-immunoreactive and neuromelanin-containing neurons throughout each tissue section in VS-ASW. We selected 3 adult brainstems with the highest tissue quality (MH001, MH004, and MH005), we counted every HSD2-immunoreactive neuron that contained a nuclear void and measured its short-axis diameter through the center of its nucleus (in μm). We used the average diameter to perform Abercrombie correction (49) and multiplied this number by the section interval (×12 for 1-in-12 series) to estimate the total number of HSD2 neurons.

For epifluorescence analysis in mouse tissue, we used QuPath (89) to count all HSD2-immunoreactive neurons and catecholaminergic NTS neurons, which are immunoreactive for the enzyme TH. We excluded 5 *Th*-IRES-Cre^–^ littermate control mice due to tears in the dorsal hindbrain, which occurred during brainstem removal and caused histological artifacts preventing analysis of the full caudal NTS.

### Statistics.

We exported continuous fluid intake records from the BioDAQ DataViewer (Research Diets) and then used Microsoft Excel to organize data and to calculate total intake volumes. After acute chemogenetic stimulation with CNO and after week-long dietary sodium deprivation, we analyzed the first 6 hours of access to 3% NaCl. For all multiday infusion protocols, we analyzed 3% NaCl and water intake in 24-hour bins. We then used GraphPad Prism to plot data and run statistical tests, which are described in figure legends. To compare dose-response effects of aldosterone on 3% NaCl and water intake, we calculated AUC across 9d following osmotic minipump implantation and then used 1-way ANOVA followed by Tukey’s multiple-comparison correction. To assess the effect of Cre-conditional neuronal ablation on aldosterone-induced intake of 3% NaCl and water, CNO effects on sodium and water intake, and sodium and water intake induced by sodium deprivation, we used unpaired, 2-tailed *t* tests. We also used unpaired, 2-tailed *t* tests to compare immunolabeled cell counts between individual groups of experimental and control mice and to compare sodium, copeptin, and glucose measurements between aldosterone-infused and control mice. To compare average daily water intake and average daily urine output between aldosterone- and vehicle-infused mice with restricted and unrestricted water access in metabolic cages, we used repeated-measures 2-way ANOVA. All results are expressed as mean ± SD. We considered *P* < 0.05 statically significant.

### Study approval.

All procedures involving animals were conducted in accordance with the guidelines of the IACUC at the University of Iowa (protocol nos. 00720011, 3072011, and 3102343). Human tissue procurement protocols were reviewed by the University of Iowa’s IRB and determined not to represent patient research under the Revised Common Rule. Consent for research tissue donation was obtained from the next of kin by the University of Iowa Department of Pathology in accordance with federal and Iowa law. In accordance with Iowa law, no tissue from elective terminations was used for this project.

### Data availability.

The data that support the findings of this study are in the Supporting Data Values file and are also available from the corresponding author upon reasonable request.

## Author contributions

MH supplied human and porcine tissue, and LP performed immunohistology, in situ hybridization, and slide-scanning microscopy, as well as morphological analysis of human HSD2 neurons and early draft illustrations. SG performed all mouse experiments. SG collected, organized, and analyzed data. MCM performed chemogenetic CNO infusion experiments with SG and with supervision from JMR. CJAM counted cells in mouse tissue. JCG supervised the project, planned experiments with SG, and analyzed all results with SG. SG and JCG drafted and edited the manuscript and figures together. All authors read, commented on, and approved the final manuscript.

## Supplementary Material

Supplemental data

Supporting data values

## Figures and Tables

**Table 3:**
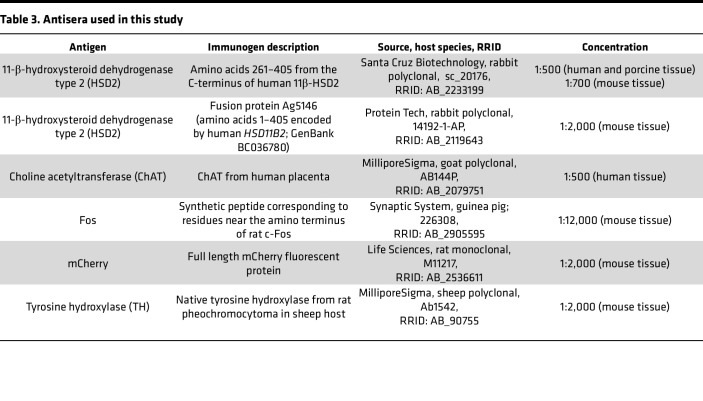
Antisera used in this study

**Table 2:**
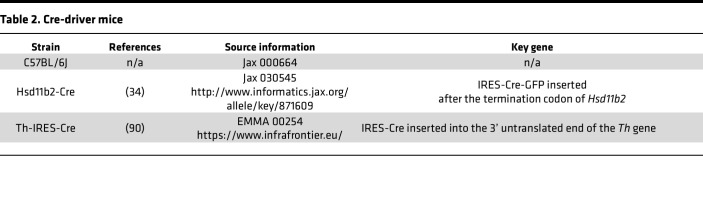
Cre-driver mice

**Table 1:**
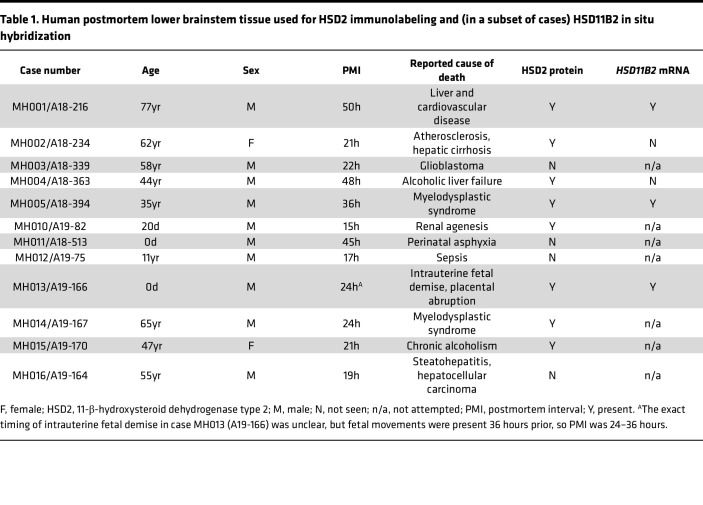
Human postmortem lower brainstem tissue used for HSD2 immunolabeling and (in a subset of cases) HSD11B2 in situ hybridization

## Version 1 10/24/2024

In-Press Preview

## Version 2 12/06/2024

Electronic publication
