# Supplementary material for: Aldosterone-induced salt appetite requires HSD2 neurons
Source: JCI Insight. 2024 Dec 6;9(23):e175087. doi: 10.1172/jci.insight.175087 (PMC11623954; doi:10.1172/jci.insight.175087)
Supplement: Supplemental data [file jciinsight-9-175087-s188.pdf]

Supplemental Figures and Legends

Supplemental Figure 1. HSD2 neurons in several mammalian species have a similar location, distribution, and appearance. (A) HSD2-immunoreactive neurons in rat NTS (brown DAB immunohistochemical labeling for HSD2 in a horizontal tissue section, with thionin counterstain), as described in (29). (B) HSD2-immunoreactive neurons in a mouse from this study (inverted grayscale image of Cy5 labeling; coronal tissue section through the caudal medulla of an aldosterone-infused mouse). (C) HSD2-immunoreactive neurons in pig NTS (NiDAB-immunohistochemical labeling in a coronal tissue section through the medulla). (D) HSD2-immunoreactive neurons in human NTS (inverted grayscale image of Cy3 labeling in a transaxial tissue section through the caudal medulla of case MH010, also shown in Figure 1E). Scale bar in each panel is 100 μm.

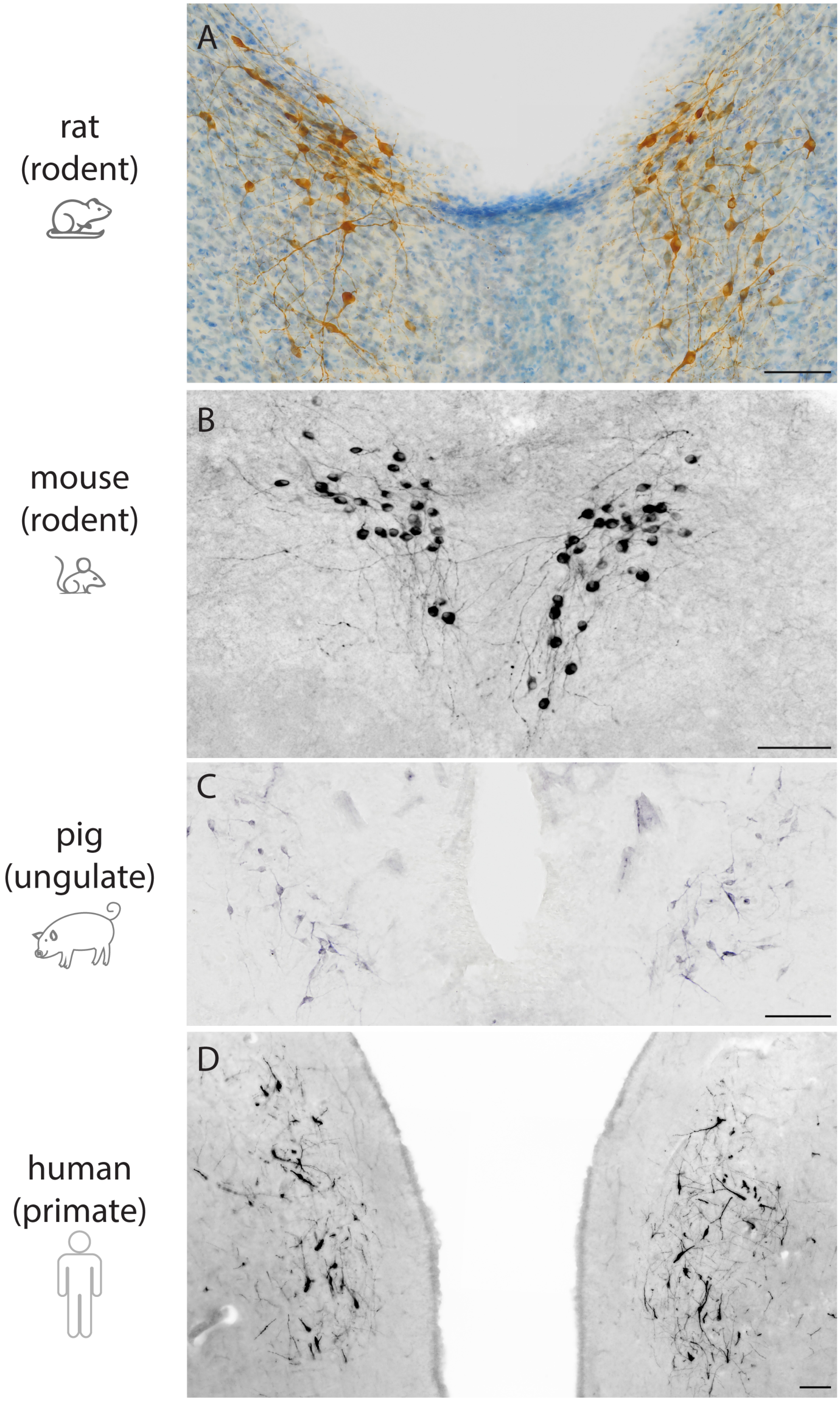
A
rat
(rodent)
B
mouse
(rodent)
C
pig
(ungulate)
D
human
(primate)

Supplemental Figure 2. Water intake, urine output, and blood measurements during s.c. infusion of aldosterone (1000 ng/h) or vehicle. (A-B) Daily and total water intake in mice treated with s.c. aldosterone or vehicle and given *ad libitum* access to water. Aldosterone increased total water intake ($p<0.0001$). (C-D) Daily and total urinary volume in mice treated with s.c. aldosterone or vehicle and given *ad libitum* access to water. Urine output was higher in animals treated with aldosterone ($p=0.0055$). (E) Scatterplot compares daily water intake and urine output volumes across all mice treated with s.c. aldosterone or vehicle and given *ad libitum* access to water. (F-G) Daily and total water intake in mice treated with s.c. aldosterone or vehicle and during and after mild water restriction (5 mL/d for 7d). Water intake increased in aldosterone-infused mice only when provided unrestricted access to water on days 8–10 (aldosterone vs. vehicle; $p = 0.0014$; aldosterone d1-7 vs. aldosterone 1-7d. vs. aldosterone 1-8d; $p = 0.0025$). (H-I) Daily and total urinary volume in mice treated with s.c. aldosterone or vehicle during and after mild water restriction (5 mL/d for 7d). There was no increase in urine output in the period that water is restricted (blue shade; $p > 0.9999$). When water intake increased in aldosterone-infused mice (d8–10), urine output increased as well (aldosterone 1-7d vs. 8-10d; $p =0.0025$; aldosterone 8-10d vs vehicle 8-10d, $p = 0.0014$). (J) Scatterplot compares daily water intake and urine output volumes across all mice treated with s.c. aldosterone or vehicle and during and after mild water restriction (5 mL/d for 7d). (K) Plasma sodium (mEq/L), (L) plasma copeptin (ng/dL), and (M) blood glucose (mg/dL) were not different between s.c. aldosterone- and vehicle-infused mice ($p=0.9523$; $p=0.0907$; $p=0.1571$, respectively).

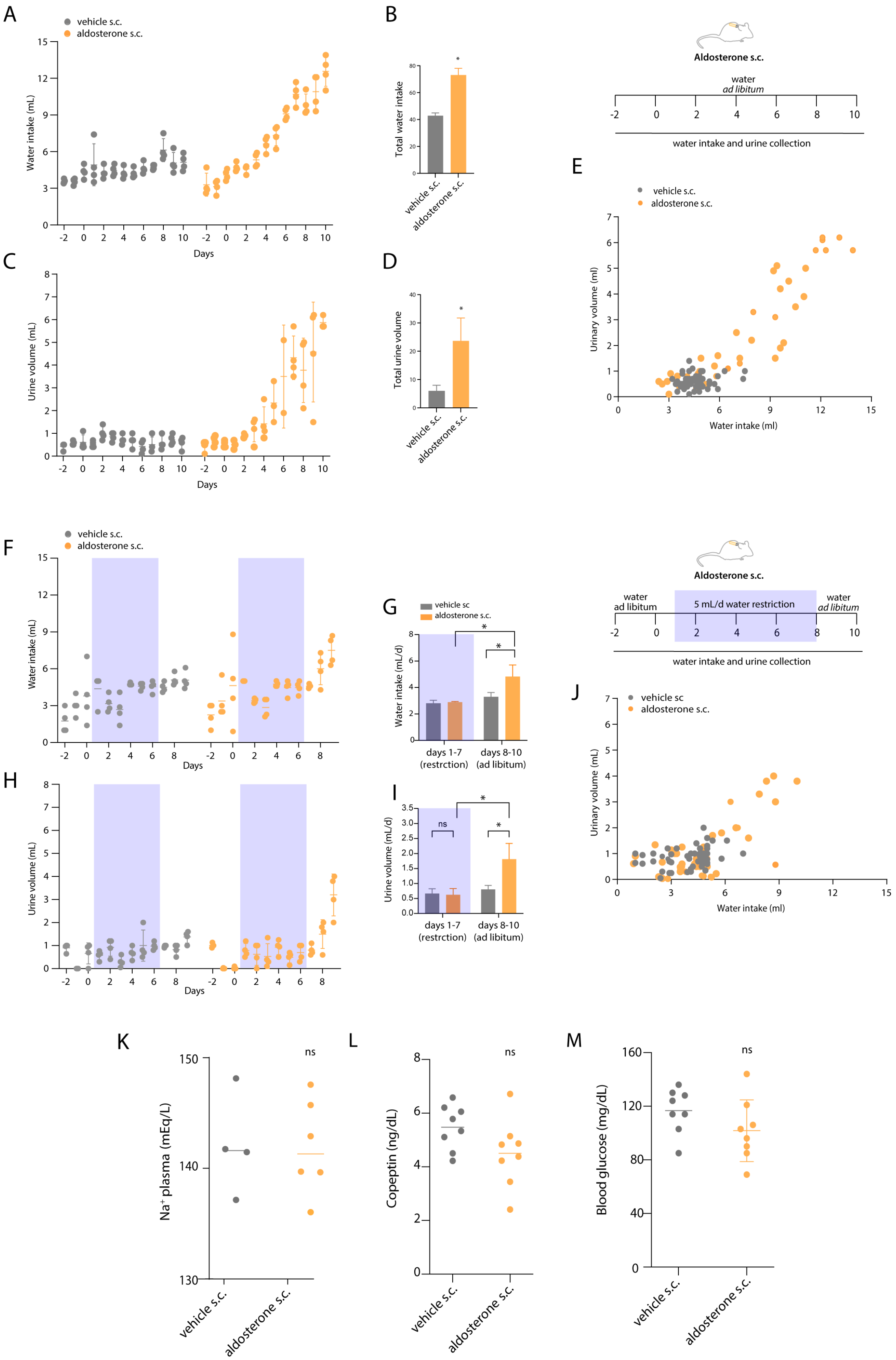

A
vehicle s.c.
aldosterone s.c.
Water intake (mL)
Days
B
Total water intake
vehicle s.c.
aldosterone s.c.
*
Aldosterone s.c.
water
ad libitum
water intake and urine collection
C
Urine volume (mL)
Days
D
Total urine volume
*
E
Urinary volume (ml)
Water intake (ml)
F
G
vehicle sc
aldosterone s.c.
Water intake (mL/d)
days 1-7
(restrction)
days 8-10
(ad libitum)
5 mL/d water restriction
water
ad libitum
H
I
Urine volume (mL/d)
ns
J
K
Na+ plasma (mEq/L)
L
Copeptin (ng/dL)
M
Blood glucose (mg/dL)
